# Pedicled iliac bone flap grafting in the treatment of late presentation Legg–Calvé–Perthes disease

**DOI:** 10.3389/fsurg.2023.926109

**Published:** 2023-03-29

**Authors:** Yong-bing Xiao, Wei Du, Pan-feng Wu, Li-ming Qing, Fang Yu, Ju-Yu Tang

**Affiliations:** ^1^ Department of Orthopedics, Xiangya Hospital, Central South University, Changsha, China; ^2^ Department of Rehabilitation Medicine, Xiangya Hospital, Central South University, Changsha, China

**Keywords:** Legg–Calvé–Perthes disease, pedicled iliac bone flap, surgical technique, late presentation, children

## Abstract

**Background:**

Legg–Calvé–Perthes disease (LCPD) is a juvenile form of ischemic femoral head osteonecrosis affecting children. The lack of effective and timely treatment results in severe sequelae in children (especially older ones). Although LCPD has been widely studied, little is known about its etiology. As a result, its clinical management is still challenging. This study will investigate the clinical and radiological results of patients older than 6 years and treated with pedicled iliac bone flap grafting for LCPD.

**Materials and methods:**

A total of 13 patients (13 hips) with late presentation of LCPD were treated with pedicled iliac bone flap grafting. Of the 13 patients, 11 were male and 2 were female. The average age of the patients was 8.4 years (range 6–13). Preoperational radiographs and pain scores were analyzed for lateral pillar classification and the Oucher scale. The final follow-up radiograph was classified using a modified Stulberg classification. Limping, extremity length inequality, and range of motion were clinically assessed.

**Results:**

The average follow-up of the patients was 70 months (range 46–120). During the surgery, seven hips were found to be lateral pillar grade B, two were grade B/C, and four were grade C. In the final examination, 12 hips were evaluated as good (Stulberg class I or II) and one as medium (Stulberg class III). There was limb shortening in one patient who was Stulberg class III. There was a significant difference between the preoperational and postoperational radiographic values and the Ocher scale, regardless of the surgical staging (*P *< 0.05).

**Conclusions:**

Pedicled iliac bone flap graft can treat LCPD accompanied by pain and lateral pillar stage B, B/C, and C in children over 6 years.

**Level of Evidence:**

Level IV—case series.

## Introduction

Legg–Calvé–Perthes disease (LCPD) was first described by Legg, Waldenström, Calvé, and Perthes in 1910. They stated that LCPD is a juvenile form of ischemic femoral head osteonecrosis affecting children between 2 and 14 years. Studies have reported that the incidence of LCPD varies between 0.4/100,000 and 29.0/100,000 in children younger than 15 years (1, 2). The symptoms of LCPD include lameness and localized pain in the hip, radiating to the thigh and knee. Nonetheless, limitations of abduction and internal rotation are also common. Although studies have shown that obesity, smoking exposure, and childhood deprivation are strongly correlated with the etiology of LCPD, its pathogenesis is still unknown (3, 4). Legg–Calvé–Perthes disease has been divided into four radiographic stages: initial or necrosis phase, fragmentation phase, reossification phase, and final phase (4, 5).

Although LCPD is a self-limited disease, young adults may develop onset arthritis, requiring hip replacement if left untreated (6–8). Studies have shown that the operative treatments (osteotomy of the femur or pelvis), alternatives for conservative methods, have good results among patients who are 6 years or older at the onset of LCPD. However, the effectiveness of these treatments in achieving a spherical femoral head is modest (9–11). Furthermore, osteotomy of the femur or pelvis has several disadvantages, including increased pressure on the hip joint capsule and unequal limb length. These disadvantages result in femoral head necrosis and make hip arthroplasty more difficult in failure cases (7, 11–14). Therefore, it is important to develop more effective treatments to prevent femoral head deformity.

Clinical researchers have confirmed that, unlike other bone flaps, the free or pedicled vascularized iliac bone flap has a better osteogenic ability, more abundant vascularity, and better remodeling effect on the shape of the femoral head. Our researchers stated that these features are an important surgical option for adult femoral head necrosis (15, 16). As a result, it was hypothesized that pedicled iliac bone flap transfer could prevent the development of LCPD and restore the femoral head sphericity. In this study, patients with LCPD were treated using pedicled iliac bone flap grafting to determine whether pedicled iliac bone flap based on deep circumflex iliac vessel grafts could result in good femoral head shape and favorable clinical outcomes during short-term follow-up.

## Materials and methods

### Patients

In total, 13 patients (13 hips) with late presentation LCPD treated using pedicled iliac bone flap graft at our hospital from 2007 to 2016 were included in this study. This study was approved by the Ethics Committee of Xiangya Hospital. A written informed consent was obtained from the legal guardian of the minor for the publication of any potentially identifiable images or data included in this article. Patients were included in this study if they (1) had LCPD with varying degrees of pain that did not respond to conservative treatment (i.e., no treatments, drugs, physiotherapy, weight relief with crutches, traction, or bed rest) for at least 6 months; (2) were 6 years and older without cognitive dysfunction; (3) had no history of hip infection or hip surgery; and (4) were in stage B, B/C, and C according to the lateral pillar staging.

Of the 13 patients, 11 were male (11 hips) and 2 were female (2 hips) with an average age of 8.4 years (range 613). Ten patients had right hip involvement, and three had left hip involvement. The average follow-up was 70 months (range 46–120). In this study, an x-ray was performed at 1, 3, 6, 9, and 12 months. After that, an x-ray was performed every 6 months postoperatively to evaluate the osseous healing and the sphere shape of the femoral head. Two independent orthopedic surgeons assessed all patients using the lateral pillar classification before the surgery. The Oucher scale is considered the most effective and commonly used self-reported measure for pain in children aged 3–12 years (17). In this study, we used it to assess pain before surgery and at the last follow-up. The radiographic outcome during the final follow-up was assessed according to the modified Stulberg classification proposed by Herring et al. (10). In the Stulberg classification, Stulberg class I or II is considered good, Stulberg class III is intermediate, and Stulberg class IV or V is a poor result. Good and intermediate outcomes are regarded as successful. All patients were clinically assessed for limping, extremity length inequality, and range of motion.

### Surgical procedure

**Figure 1:**
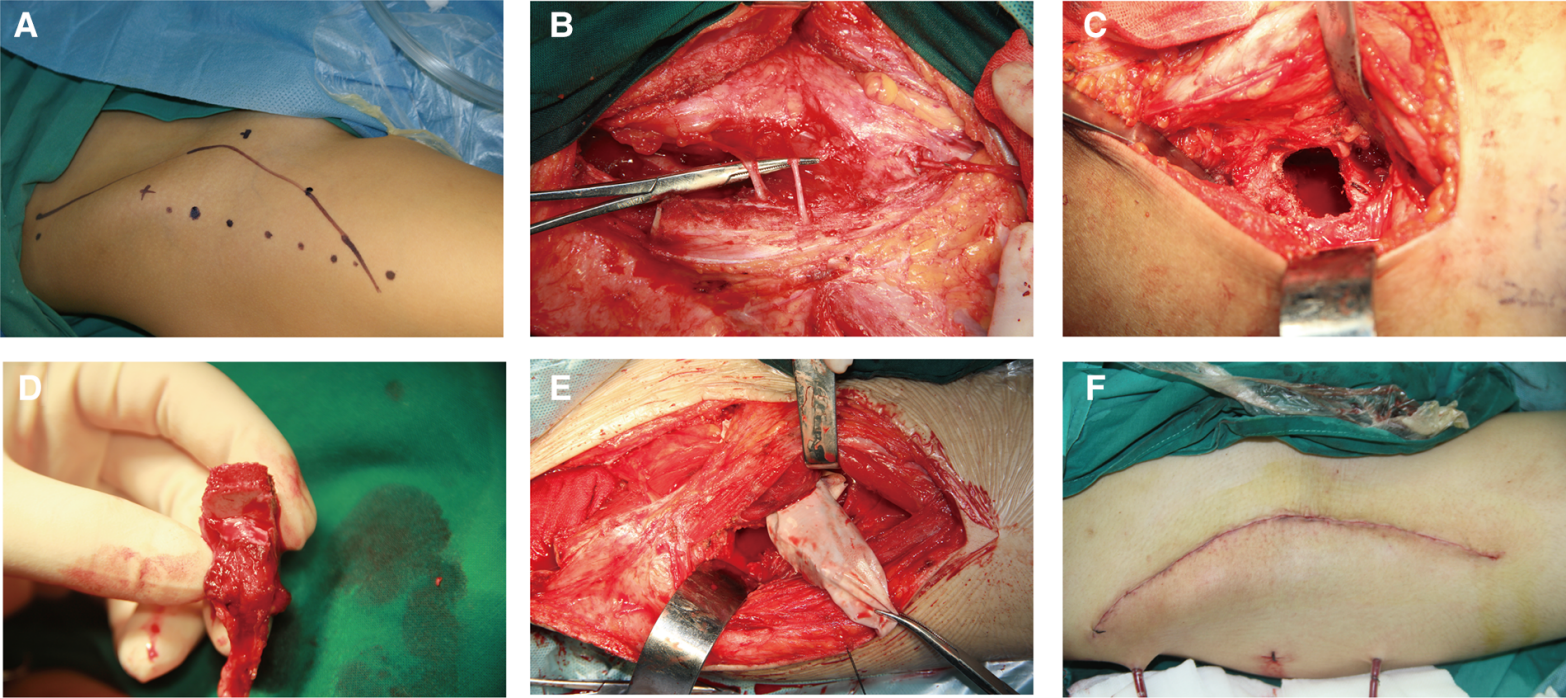
Surgical step. (**A**) A Smith–Petersen incision was designated through the groin to the thigh to expose the donor site for harvesting a pedicled iliac bone flap and the necrotic femoral head. (**B**) The skin and subcutaneous tissue were dissected, and the lateral femoral cutaneous nerve was exposed and protected. (**C**) The bone window was designed on the proximal femoral neck. (**D**) The pedicled iliac bone flap based on the deep circumflex iliac artery was harvested and confirmed by punctate bleeding points from the osteotomy side. (**E**) The transferred pedicled iliac bone flap was put in a sterile glove and transported through a tunnel under the rectus femoris and inguinal ligament. (**F**) The incision was closed layer by layer.

After general anesthesia, the patients were placed in a supine position, and all procedures were performed by one surgery team. A standard Smith–Petersen skin incision was made. The incision began at the middle of the iliac crest, continued anteriorly to the anterior superior iliac spine, and distally and slightly laterally 10 cm–12 cm (Figure 1A). The skin and subcutaneous tissue were dissected, and the lateral femoral cutaneous nerve was exposed and protected (Figure 1B). A blunt dissection was performed in the intermuscular space between the sartorius and rectus femoris, and the ascending branch of the lateral circumflex femoral artery and the reflex head of the rectus femoris were ligated. After retracting the tensor fasciae latae, sartorius, and rectus femoris, the hip joint capsule was exposed. Subsequently, the hip joint capsule was dissected in the shape of a cross to expose the femoral head on the anterior side. Then, the bone window was designed on the femoral neck (about 2 cm × 1.2 cm). Finally, the partial capsule and the bone window of the femoral head were removed (Figure 1C), and necrotic lesions were debrided thoroughly under direct vision. The articular cartilage surface, the thin layer of subchondral bone, and an epiphyseal plate were preserved. The iliac bone flap was obtained as described previously (15, 16). The vascularized iliac crest bone flap (typically 3 cm × 2 cm × 1.2 cm) was harvested from the iliac crest pedicled with deep iliac circumflex vessels. The blood supply of the harvested iliac crest bone flap was confirmed using punctate bleeding points from the osteotomy side (Figure 1D). The harvested bone flap was put in a plastic bag or sterile glove and transferred to the bone window of the femoral neck, passing through a tunnel under the rectus femoris and inguinal ligament. The bone flap was then planted into the defect of femoral head necrotic lesions and adequately trimmed. After a successful trial, the cancellous bone from the iliac crest was implanted into the femoral head, and the iliac bone flap was inserted into the bone groove (Figure 1E). Homeostasis was then performed. If no bleeding was detected in the wound, drainage was placed, and the incision was closed layer by layer (Figure 1F).

### Postoperative management

The most suitable cephalosporins were used as prophylactic antibiotics during the perioperative period (48–72 h), and papaverine prevented vasospasm 3–7 days postoperatively. Abduction and internal rotation plaster fixation were performed for 3 weeks. One to three months after surgery, postoperative non-weight-bearing rehabilitation training was performed in bed. Subsequently, according to the results of anteroposterior (AP) pelvic radiography and under the guidance of physical therapists, rehabilitation exercises with partial and full weight bearing were performed under the guidance of physical therapists until the return to normal life and study. However, strenuous exercise should be avoided for at least 1 year.

### Statistical analysis

Statistical analyses were performed using SPSS 22.0. The Mann–Whitney *U* test was used to analyze data. A *P-*value of less than 0.05 was considered statistically significant.

## Results

In this study, 13 patients (13 hips) accepted pedicled iliac bone flap grafting to treat late presentation LCPD. During surgery, seven hips were in the lateral pillar grade B, two were in grade B/C, and four were in grade C. The diagnosis age, side, sex, bone flap area, operation time, complications, preoperative and postoperative radiological staging of the patients, and Oucher scale are summarized in Table 1.

**Table 1:** Clinic features distribution of LCPD patients treated with pedicled iliac bone flap grafting.

Patient	Gender	Diagnosis age (year)	Side	Bone flap area (cm)	Operation time (min)	Follow (m)	Complications	Lateral pillar	Stulberg class	Pre-Oucher scale	Post-Oucher scale
1	Male	7	R	3 × 2 × 1	195	82	None	B	I	4	0
2	Male	8	R	2 × 2 × 1.5	165	88	None	C	III	6	2
3	Male	6	L	2 × 1 × 1	165	46	None	B	II	5	1
4	Male	10	R	3 × 1.5 × 1	170	120	None	B/C	II	6	1
5	Female	6	R	4 × 3 × 1.5	190	88	None	B	II	5	1
6	Male	8	R	3 × 2 × 2	175	70	None	C	II	6	2
7	Male	6	R	2 × 2 × 1	170	67	None	B	II	5	1
8	Female	9	R	4 × 2 × 1.5	180	49	None	B	I	4	1
9	Male	13	R	4 × 2 × 1	185	68	None	B/C	II	6	2
10	Male	8	L	3 × 2 × 1	160	52	None	C	II	6	1
11	Male	13	L	4 × 3 × 1.5	180	65	None	B	II	4	0
12	Male	7	R	2 × 2 × 1	160	65	None	B	II	4	0
13	Male	8	R	3 × 2 × 2	178	56	None	C	II	5	1

LCPD, Legg–Calvé–Perthes disease.

During the final follow-up (average 83 ± 37 months), the patients were evaluated using the Stulberg classification. Of the 13 hips, 12 were evaluated as good (Stulberg I or II) and 1 as intermediate (Stulberg III). In this study, no hip was evaluated as poor. Therefore, all patients had an acceptable outcome (Stulberg I–III).

The lateral pillar grade was significantly different between patients with lateral pillar B or B/C and patients with grade C during the final follow-up period (*P < *0.05, Table 2). Patients with lateral pillar grade B and B/C achieved good results. In addition, good outcomes were obtained in 75% of patients with lateral pillar grade C.

**Table 2:** Stulberg classification distribution in the final follow-up according to the preoperative lateral pillar classification.

Stulberg class	I	II	III
Lateral pillar B	2 (29%)	5 (71%)	0%
Lateral pillar B/C	0%	2 (100%)	0%
Lateral pillar C	0%	3 (75%)	1 (25%)

**Figure 2:**
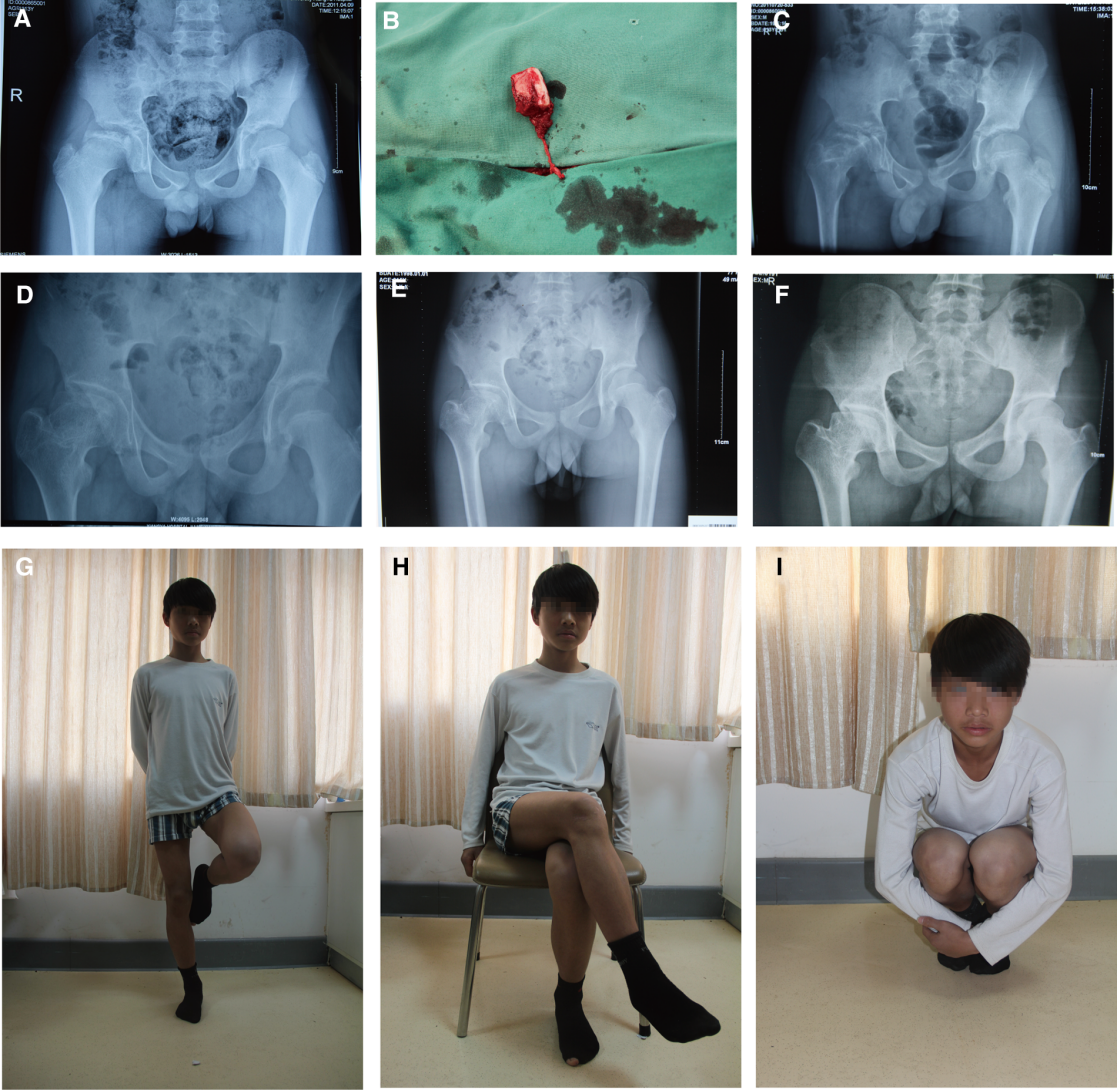
A 13-year-old boy underwent a pedicled iliac bone flap based on deep circumflex iliac vessels to treat LCPD of the right side (lateral pillar grade B/C). (**A**) Preoperative x-ray film. (**B**) The pedicled iliac bone flap was harvested. Postoperative AP x-rays at (**C**) 3 months, (**D**) 17 months, (**E**) 24 months, and (**F**) 69 months showed no collapse of femoral heads or narrowing of the hip joint spaces. Hip (**G**) extension, (**H**) external rotation, and (**I**) flexion showed good recovery. LCPD, Legg–Calvé–Perthes disease; AP, anteroposterior.

**Figure 3:**
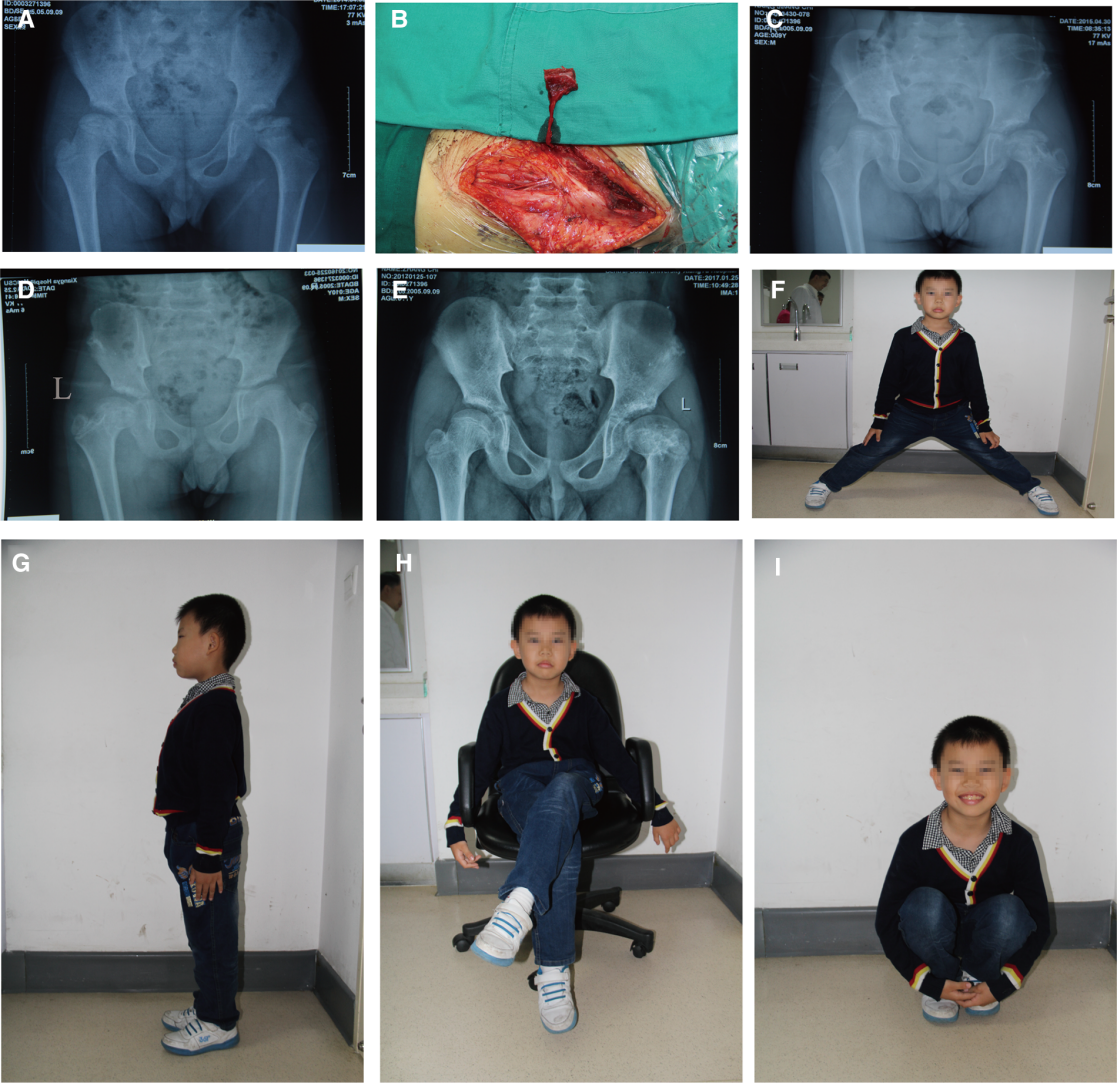
An 8-year-old boy underwent a pedicled iliac bone flap based on deep circumflex iliac vessels to treat LCPD of the left side (lateral pillar grade C). (**A**) Preoperative x-ray film. (**B**) The pedicled iliac bone flap was harvested. Postoperative x-rays at (**C**) 12 months, (**D**) 24 months, and (**E**) 36 months showed no collapse of femoral heads or narrowing of the hip joint spaces. Hip (**F**) abduction, (**G**) Extension, (**H**) external rotation, and (**I**) flexion showed good recovery. LCPD, Legg–Calvé–Perthes disease.

Clinically, all 13 patients got primary healing with no complications. The average operation time was 175 min (range 160–195). Postoperative imaging revealed that the 13 patients received a complete bone union and improved femoral head shape. The postoperative Oucher scale (1.0 ± 0.7) was lower than the preoperative Oucher scale (5.1 ± 0.9) (*P < *0.05, Table 1). All patients showed varying degrees of improvement in limping and extremity length inequality. One patient had abduction function restriction and was classified as Stulberg class III. The patient presented with a mild limp and a little pain when acting. Preoperative and postoperative radiographs of two patients are shown in Figures 2, 3.

## Discussion

The formation and progression of LCPD are mainly caused by the interruption of blood supply to the femoral head, leading to extensive ischemic cell death in the epiphyseal marrow and the trabecular bone (4). Although LCPD has been widely studied, there is no standardized and optimal treatment protocol for patients with LCPD. However, pedicled iliac bone flap grafting has been used to treat advanced LCPD. Studies have reported that the pedicled iliac bone flap grafting restores the blood supply to prevent osteonecrosis remodeling the sphere shape of the femoral head, and preventing further collapse and secondary osteoarthritis (14–16).

This study demonstrated that a spherical femoral head in Herring B, B/C, and C pediatric patients can be achieved using pedicled iliac bone flap grafting. Furthermore, we achieved a good outcome in 100% of patients in Herring grade B and B/C and 75% of patients in Herring grade C. Only one LCPD patient with a Herring grade C was classified as Stulberg class III due to early weight-bearing activity. In addition, these outcomes were achieved without surgical complications and serious leg length differences. In addition, a lower pain score was measured during the final follow-up. The apparent relief in pain may be related to the removal of dead tissue from the necrotic area of the femoral head during surgery.

Therefore, the pedicled iliac bone flap graft is a promising alternative treatment protocol for patients with LCPD and has several advantages. First, the synovium and other pathological tissues in the articular cavity can be removed during surgery and fresh cancellous bone implanted to maintain and restore the shape of the femoral head and increase the inclusiveness of the femoral head within the acetabulum. Second, the necrotic bone and granulation tissue can be removed under direct vision while performing fenestration and decompression on the femoral head. This method promotes new bone regeneration and blood vessel growth and relieves the intraosseous and intracapsular pressure of the hip joint, which relieves pain. Third, the planted vascularized iliac bone flap based on the deep circumflex iliac artery (DCIA) provides an additional blood supply for the affected femoral head and has a strong osteogenic capacity and healing ability. Studies have reported that DCIA acts as a bony support preventing the femoral head from collapsing. Therefore, using a pedicled iliac bone flap graft to treat patients with LCPD may temporarily delay or prevent the progression of osteoarthritis (18, 19). Finally, this surgical procedure can be completed with one incision in a convenient position, there is no microvascular anastomosis, and it causes minimal trauma to the femoral neck and trochanter; thus, this surgical option is suitable for clinical application and surgical failure does not affect the patient's future hip replacement.

The precautions of our surgical method in treating LCPD are as follows: (1) thorough debridement of the femoral head necrotic area should be performed; (2) the femoral head window should be properly sized to ensure that the iliac bone flap is firmly embedded and the iliac crest bone and periosteum should be joined to avoid the interruption of blood supply to the iliac crest bone flap; (3) the lateral femoral cutaneous nerve passing over the sartorius 2–3 cm distal to the anterior superior iliac spine should be protected well by retracting it to the medial side; (4) the dissection of DCIA should be performed carefully and precisely to avoid damaging the blood supply of the iliac crest bone flap; (5) closure of the incision should be performed by hierarchical anatomical correspondence to avoid a hernia; and (6) at least 3 weeks of postoperative immobilization in plaster or brace is required to avoid vascular distortion and bone flap loosening. The intensity of rehabilitation training and the timing of getting out of bed should be based on the x-ray results and the repair of the necrotic area. Too long or too short braking time is not conducive to remodeling a soft femoral head into a spherical shape by the acetabular socket and the recovery of hip function.

This study has several limitations. First, we routinely used x-ray to observe the healing of the bone flap after surgery and examination results to speculate the blood supply in the bone flap. Although CT or MRI can also be used to evaluate the survival of the iliac flap, there are no intuitive and effective methods to observe the blood supply of the deep circumflex iliac vascular pedicle in the iliac flap. Second, this procedure affects the development of the hip joint. Although the procedure prevents collapse of the femoral head, it cannot restore it to a completely normal shape. Therefore, it is difficult to make the head–acetabulum relationship completely normal. Studies have also reported that this procedure damages epiphyses, leading to limb shortening after skeleton maturity (4, 8, 11). In this case, high insoles can balance the unequal length of the lower limbs. Third, this study used a small number of patients and lacked a control group. Thus, the findings may be limited with low reliability. Although longer-term follow-up is required, we believe that the pedicled iliac bone flap graft is a good surgical option for patients with Herring grades B, B/C, and C accompanied by pain after 6 years of age.

## Data Availability

The raw data supporting the conclusions of this article will be made available by the authors, without undue reservation.
